# Quantitative Analysis of Blended Asian Lacquers Using ToF–SIMS, Py–GC/MS and HPLC

**DOI:** 10.3390/polym13010097

**Published:** 2020-12-29

**Authors:** Hye Hyun Yu, Jung-Ah Lim, Seung Wook Ham, Kang-Bong Lee, Yeonhee Lee

**Affiliations:** 1 Advanced Analysis Center, Korea Institute of Science and Technology, Seoul 02792, Korea; 091615@kist.re.kr; 2 Department of Chemistry, Chung-Ang University, Seoul 06974, Korea; swham@cau.ac.kr; 3 Post-Silicon Semiconductor Institute, Korea Institute of Science and Technology, Seoul 02792, Korea; jalim@kist.re.kr; 4 National Agenda Research Division, Korea Institute of Science and Technology, Seoul 02792, Korea; leekb@kist.re.kr

**Keywords:** blended lacquer, quantitative analysis, ToF–SIMS, Py–GC/MS, HPLC

## Abstract

Asian lacquer is a special polymeric material tapped from lacquer trees. The tree’s sap is a complex mixture of compounds, such as catechol lipids, polysaccharides, glycoproteins, enzymes, and water. Researchers have not yet quantitatively analyzed blended lacquers. We evaluated the compositions of Japanese and Vietnamese lacquers, and blends of the two, using time-of-flight secondary ion mass spectrometry (ToF–SIMS), pyrolysis–gas chromatography/mass spectrometry (Py–GC/MS), and high-performance liquid chromatography (HPLC). ToF–SIMS provided quantitative results for blended lacquers; provided structural information on polymeric lacquer films; and indicated the presence of dimers of urushiol–urushiol, urushiol–laccol, and laccol–laccol derivatives. We used Py–GC/MS and HPLC to obtain linear calibration curves. The specific peak intensity was a linear function of the ratio of Japanese to Vietnamese lacquer in the blends. For an unknown mixture, all three techniques gave essentially the same results. These quantitative methods will be useful for improving the physical properties of polymeric lacquer films, and evaluating the lacquer quality in industry and historic conservation.

## 1. Introduction 

Natural Asian lacquers have been widely used as coating materials and binding media in Asia for thousands of years because they provide highly useful properties, such as brilliance, beauty, and durability [1,2,3,4,5]. Multiple and complex layers of lacquer are used to decorate the surfaces of screens, boxes, dishes, cabinets, and small objects, imparting a distinctive appearance that is also pleasantly to touch. Furthermore, researchers must discriminate the type of lacquer for optimum conservation and restoration of ancient lacquer remains [6,7,8,9,10,11]. One collects Asian lacquer sap mainly from three types of trees from the *Anacardiaceae* family. *Toxicodendron vernicifluum* grows in Japan, Korea, and China; its main lipid component is urushiol. The catecholic lipid component in *Toxicodendron succedaneum* from Taiwan and Vietnam is laccol, whereas it is thitsiol in *Gluta usitata* from Thailand and Myanmar [12,13,14,15,16]. It is worth noting that some form of pretreatment is required in order to convert the raw tree saps to a material suitable for application. In the drying process of lacquer sap, the major lipid components such as urushiol, laccol, and thitsiol are catalyzed by laccase to generate catechol radicals for polymerization [4,13].

Researchers have used various analytical techniques, such as high performance liquid chromatography (HPLC), Fourier transform infrared spectroscopy, nuclear magnetic resonance spectroscopy, thermal analysis, and gas chromatography/mass spectrometry (GC/MS) to characterize the structures and chemical compositions of Asian lacquers [17,18,19,20,21]. However, because lacquer films have complex molecular compositions and are cross-linked structures that do not readily dissolve in solvents, they are difficult to characterize by conventional analytical techniques.

Coupling pyrolysis to direct inlet MS, evolved gas analysis ion attachment MS, and GC/MS is useful for analyzing lacquers that have inherently complex polymeric networks after curing [14,22,23,24,25]. Py–GC/MS is a common technique for analyzing and identifying Asian lacquers. In particular, researchers have applied Py–GC/MS with tetramethyl ammonium hydroxide as a derivatization agent for the characterization of the lacquer materials in the conservation and restoration of valuable historic objects [21,26,27].

Researchers have also identified the chemical compositions of Asian lacquers using surface techniques, such as time-of-flight−secondary-ion mass spectrometry (ToF–SIMS) and X-ray photoelectron spectroscopy [28,29,30]. These analytical techniques are useful for characterizing polymeric lacquer materials because of the minimal quantity of sample required, high sensitivity, and no sample pretreatment. Previously, we characterized lacquer specimens from Japanese, Korean, and Chinese *T. vernicifluum*; Vietnamese *T. succedaneum*; and Myanmarese *G. usitata* [31]. The ToF–SIMS results for three Asian lacquers indicated specific peaks for polymeric catechol lipids, such as urushiol, laccol, and thitsiol. In contrast, most previous studies on Asian lacquers have focused on identifying chemical compositions in accordance with the regions of production. Such research does not provide quantitative information on the type of lacquers in mixtures. 

Cultivating and collecting uncommon *T. vernicifluum* lacquer is difficult, and thus *T. vernicifluum* lacquer is very expensive. A straightforward, sensitive, and reliable quantitative lacquer analysis method would be useful for two reasons. One, it would help identify adulteration with inexpensive lacquer or lacquer analogues. Two, it would help researchers use their knowledge of the type and quantity of catechol present to improve the physical and chemical properties of mixed Asian lacquers.

In this study, we mixed Japanese (urushiol lipid) and Vietnamese (laccol lipid) lacquers. We undertook quantitative analysis of the blends by ToF–SIMS, Py–GC/MS, and HPLC. We determined the specific catechol compositions and found the possibility to apply these results toward optimizing the physical properties of natural lacquers.

## 2. Materials and Methods 

### 2.1. Materials

The raw lacquers were the saps collected from Japanese *T. vernicifluum* and Vietnamese *T. succedaneum* trees, and were purchased from Pyung Hwa Shell (Seoul, Korea). We filtered pure lacquer saps by conventional methods with Korean traditional paper. The two kinds of Asian lacquer saps were blended in various ratios (Table 1). We stirred them for 10 min at room temperature and allowed the post-stirring bubbles to dissipate for 30 min. The blended lacquer saps were coated on silicon wafers for ToF–SIMS. The thickness of the lacquer films was ≈10 µm. We dried the films for 7 d at the room temperature in a chamber with 75% relative humidity; reproduced 3×. Acetonitrile (HPLC grade) was obtained from Sigma-Aldrich Co. (St. Louis, MO, USA). Other common chemicals were the highest purity commercially available. Urushiol (3-pentadecatrienyl catechol) was purchased from Phytolab GmbH and Co. KG (Vestenbergsgreuth, Germany), and laccol (3-heptadecyl catechol) was obtained from BOC Sciences (Shirley, NY, USA). We purchased turpentine oil―the solvent―from a local supplier (Pyung Hwa Shell, Seoul, Korea).

### 2.2. Instrumentation

TOF–SIMS was performed with a TOF.SIMS 5 system from IONTOF GmbH (Münster, Germany) at the Korea Institute of Science and Technology. We used bunched mode 30-keV Bi^3+^ ions as the analysis probe with a rastered area of 100 µm × 100 µm. The target current was controlled consistently at a pulsed current of 0.6 pA. We compensated the charge on the sample surface with an electron flood gun that emitted a pulsed beam of electrons. The charge effects were controlled using a surface potential of −10 V. We used a cycle time of 100 µs, corresponding to a mass range of 0–800 *m/z*. The positive-ion mass spectra were measured to obtain information on the surface compositions for all of the blended lacquer samples. The total ion doses were below the static limit, 1 × 10^12^ ions⋅cm^−2^, to avoid accumulated damage and destroying the chemical structure of the surface layers.

For quantitative analysis of the blended lacquers, Py–GC/MS was conducted with a Multi-shot Pyrolyzer PY–2020iD (Frontier Lab, Koriyama, Japan), a 6890 N gas chromatograph (Agilent Technologies, Inc. Santa Clara, CA, USA) and a Pegasus IV mass spectrometer (LECO, St. Joseph, MI, USA). The pyrolysis temperature was 500 °C and maintained for 20 s using a platinum sample cup, which contained 0.3 mg of lacquer samples. The interface temperature was 300 °C. The GC oven temperatures were as follows: initial temperature 40 °C (2 min); then 20 °C/min, until 320 °C (14 min).

HPLC analysis was performed using Agilent Technologies 1290 infinity and 1200 series (Santa Clara, CA, USA). We conducted chromatographic separations with a YMC-Pack Pro C18 reverse-phase column (250 × 4.6 mm I.D., S-5 µm, 12 nm; YMC Co., Kyoto, Japan). The mobile phase consisted of two eluents. The eluent for urushiol (3-pentadecatrienyl catechol) was a 90:10 (*v*/*v*) mixture of acetonitrile and 0.1 vol% aqueous trifluoroacetic acid. The eluent for laccol (3-heptadecyl catechol) was a 5:95 (*v*/*v*) mixture of 0.1 vol% aqueous tetrahydrofuran and acetonitrile. Table 2 summarizes the detailed analysis parameters for ToF–SIMS, Py–GC/MS and HPLC.

## 3. Results and Discussion

### 3.1. ToF–SIMS

ToF–SIMS provides important information on the molecular structures and chemical compositions of organic and polymeric materials. We obtained positive-ion ToF–SIMS analyses of Japanese *T. vernicifluum* (Figure S1) and Vietnamese *T. succedaneum* (Figure S2) lacquer films to differentiate and quantify lacquers, over the mass range *m/z* = 1–720. In a positive-ion ToF–SIMS spectra of Japanese *T. vernicifluum* lacquer films over the mass range *m/z* = 0–100, there are oxygen-containing peaks and hydrocarbon peaks, such as C_3_H_5_^+^ (41.04), C_2_H_3_O^+^ (43.02), C_3_H_3_O^+^ (55.02), C_4_H_7_^+^ (55.06), C_6_H_5_^+^ (77.04), and C_7_H_7_^+^ (91.05). We also observed element peaks, such as Na, Mg, Al, Si, K, Ca, and Mn. As one can expect from major urushiol derivatives of Japanese *T. vernicifluum*, we observed specific aromatic fragment ions from catechol compounds, such as C_7_H_7_O_2_^+^ (123.0), C_8_H_9_O_2_^+^ (137.0), and C_10_H_13_O_2_^+^ (165.0), in the high-mass range between *m/z* = 100 and 200. In the mass range from *m*/*z* 300–370, there were pentadecyl catechol ions, such as the side chains of C_15_H_25–31_. ToF–SIMS spectra for Vietnamese *T. succedaneum* indicated laccol lipids containing dihydroxy-benzene structures bonded in the 3-position by the aliphatic carbons of C_17_H_31–35_ (Figure S2). We observed specific peaks for C_23_H_34_O_2_^+^ (342.3), C_23_H_36_O_2_^+^ (343.3), and C_23_H_38_O_2_^+^ (346.3) from heptadecyl catechols. 

**Figure 1:**
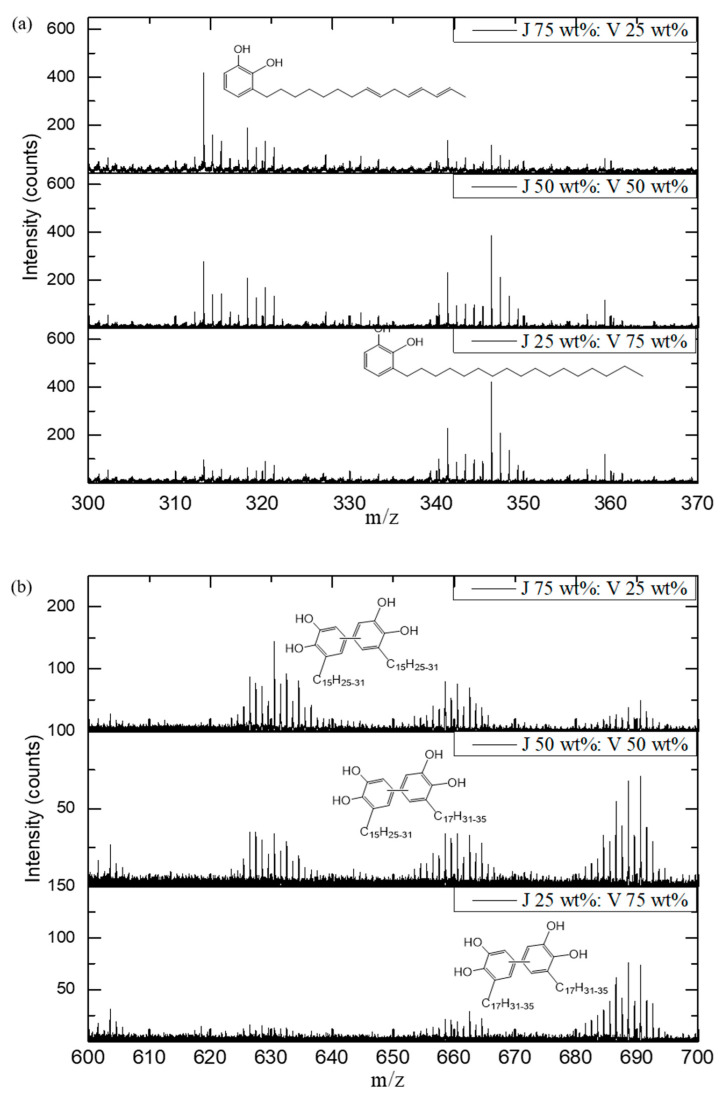
Positive-ion ToF–SIMS mass spectra of blended lacquer films of Japanese *Toxicodendron vernicifluum* and Vietnamese *T.* succedaneum in the following mass ranges: (**a**) *m/z* = 300−370; (**b**) *m/z* = 600−700.

Figure 1a shows ToF–SIMS spectra for blended lacquers of Japanese *T. vernicifluum* and Vietnamese *T. succedaneum*, in accordance with the mixing ratios. The heptadecyl catechol ion intensities at *m/z* = 342–350 increased as the portion of *T. succedaneum* lacquer increased. We also observed the high-intensity peaks of urushiol dimers that have two repeating urushiol units (C_42_H_60_O_4_; Figure 1b). These urushiol derivatives have one, two, or three double bonds. We observed laccol dimers with two repeating laccol units (C_46_H_64_O_4_^+^) in the mass range *m/z* = 684–692. In the high-mass range (*m/z* 600–700), the ToF–SIMS spectra of Japanese *T. vernicifluum* and Vietnamese *T. succedaneum* lacquer films showed urushiol–urushiol and laccol–laccol dimers, respectively. Spectra of blended lacquers indicated several molecular ions, which correspond with urushiol–laccol dimers generated by cross-coupling [32,33,34]. The polymerization mechanism in mixed lacquers of Japanese *T. vernicifluum* and Vietnamese *T. succedaneum* can be explained as follows. Laccase-catalyzed dehydrogenation of catechol radicals from urushiol occurs because of enzymatic oxidation, and catechol radicals of laccol are generated with catechol radicals of urushiol through radical transfer. Urushiol–laccol dimers are produced by a coupling reaction between these two radicals. Thus, ToF–SIMS indicates that urushiol and laccol can be copolymerized by a radical transfer of a laccase-catalyzed reaction. 

**Figure 2:**
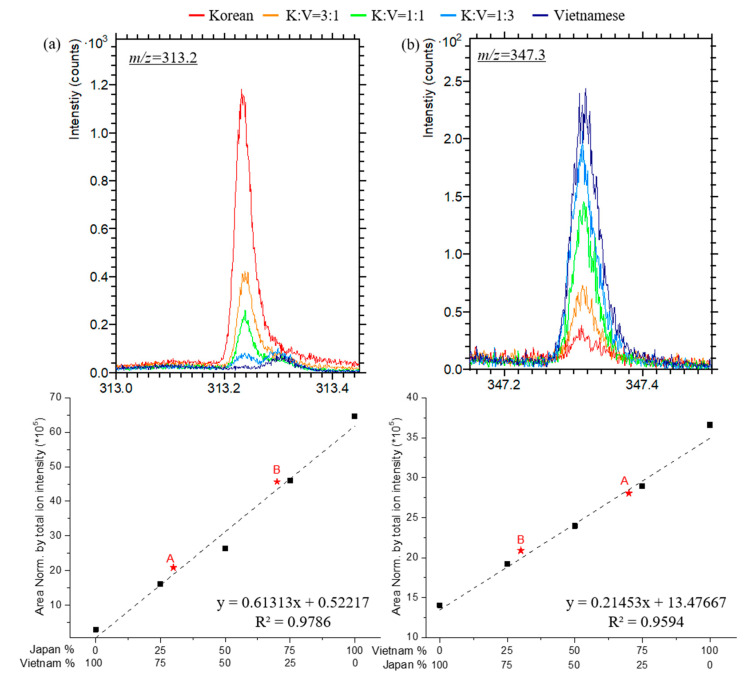
Calibration curves of the compositional ratios of blended Japanese and Vietnamese lacquer films in accordance with the detected ion species using ToF–SIMS: (**a**) urushiol ion (*m/z* = 313) and (**b**) laccol ion (*m/z* = 347).

We mixed two Asian lacquer saps in several ratios (Table 1). After the ToF–SIMS characterization of the Japanese *T. vernicifluum* and Vietnamese *T. succedaneum* lacquer films, we selected specific peaks to perform quantitative analyses. One representative peak for Japanese lacquer was pentadecyl catechol ion, C_21_H_29_O_2_^+^, at *m/z* = 313.2. We investigated the peak intensities and normalized them by using the corresponding total intensity for each spectrum. Figure 2a shows that the quantitative results obtained from the pentadecyl catechol peak are consistent with the compositional ratios of the blended lacquers. We also used another representative peak for Vietnamese lacquer―heptadecyl catechol ion, C_23_H_39_O_2_^+^, at *m/z* = 347.3―for method confirmation. The peak intensities increased as a function of the composition (Figure 2b).

### 3.2. Py–GC/MS

**Figure 3:**
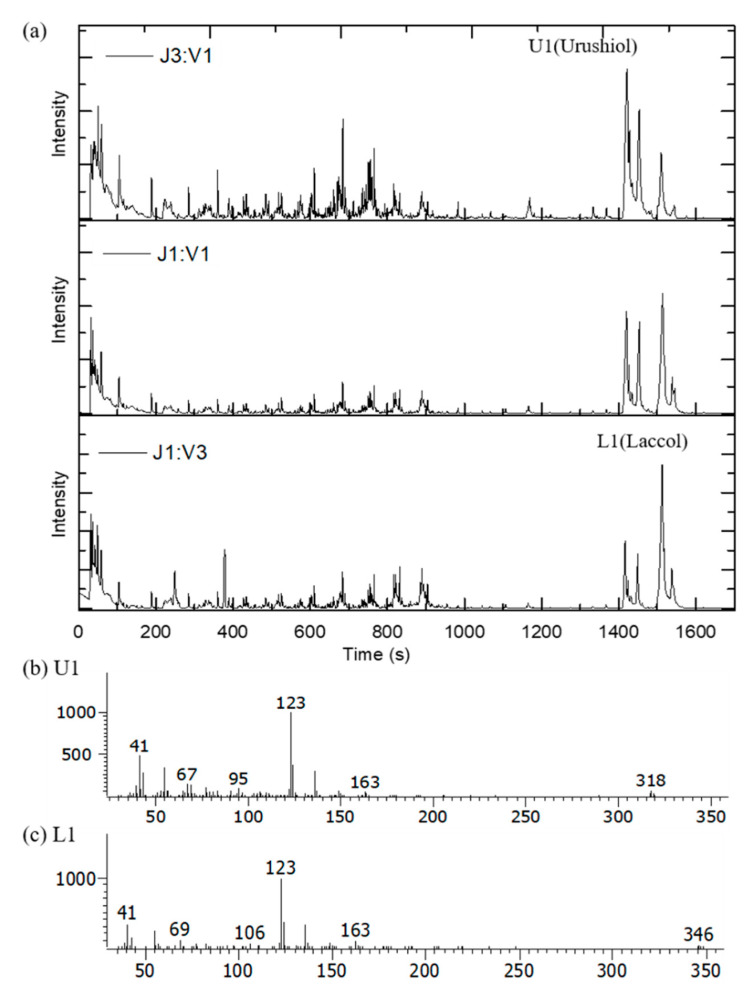
(**a**) Py–GC/MS total ion chromatogram of blended Japanese and Vietnamese lacquer films; (**b**) spectrum of peak U1; (**c**) spectrum of peak L1.

We used Py–GC/MS to compare the intensity of specific components in the pyrograms. At a pyrolysis temperature of 500 °C, we observed most of the characteristic components from the lacquer. Figure 3a shows total ion chromatograms (TICs) of Japanese and Vietnamese blended lacquers. The main pyrolytic products were aliphatic hydrocarbon components, phenol derivatives, and catechol derivatives. We clearly identified peak U1 in the Japanese lacquer TIC [Figure 3b], which indicated that 3-pentadecyl catechol is the main component of urushiol. The high-intensity peak at *m/z* = 123 corresponds to 3-methylcatechol. Alkylcatechol and alkylphenol derivatives were generated by pyrolytic cleavage of the aliphatic chains of the lacquer films. Peak L1 in the Vietnamese lacquer TIC corresponds to the 3-heptadecyl catechol (*m/z* = 346; Figure 3c). Thus, pyrolysis enables one to differentiate between the lacquer films, as shown next.

**Figure 4:**
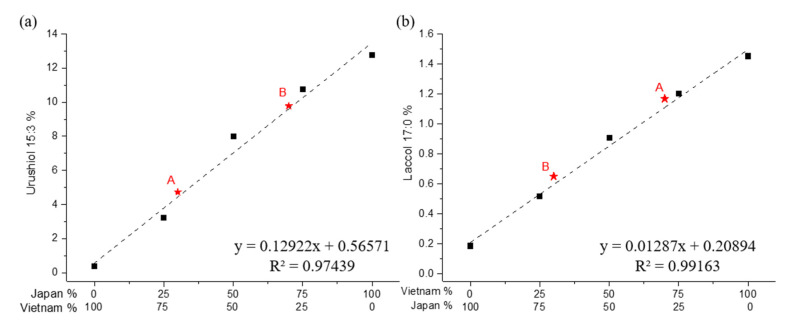
Calibration curves of the compositional ratios of blended Japanese and Vietnamese lacquer films in accordance with the detection peaks using Py–GC/MS: (**a**) urushiol peak (U1); (**b**) laccol peak (L1).

Figure 4a,b shows the intensities of peak U1 and peak L1 in accordance with the lacquer ratios, respectively. The intensity of peak U1 decreased as the proportion of Japanese lacquer decreased in the blends. The intensity of peak L1 increased as the proportion of the Vietnamese lacquer increased in the blends. The use of U1 and L1 peaks showed the quantitative results having coefficients of determination (R^2^) of 0.9944 and 0.9916, from Py–GC/MS measurements. Trend lines obtained by Py–GC/MS were superior to those obtained by ToF–SIMS. The matrix effect influences sputtering and ionization in ToF–SIMS, even though one may expect that the normalization by the total intensity would correct for differences in instrumental analysis. 

### 3.3. HPLC

Researchers commonly use HPLC to separate, identify, and quantify each component in complex mixtures. We used HPLC to determine the compositions of our lacquer blends quantitatively. Lacquer saps are complex mixtures containing, e.g., various catechol lipids, water, glycoproteins, polysaccharides, and laccase. Thus, quantitative analysis of the blended lacquers is not straightforward. We selected and used a representative marker for each lacquer―3-pentadecatrienyl catechol (Japanese lacquer) and heptadecyl catechol (Vietnamese lacquer)―as a reference (Table 2).

**Figure 5:**
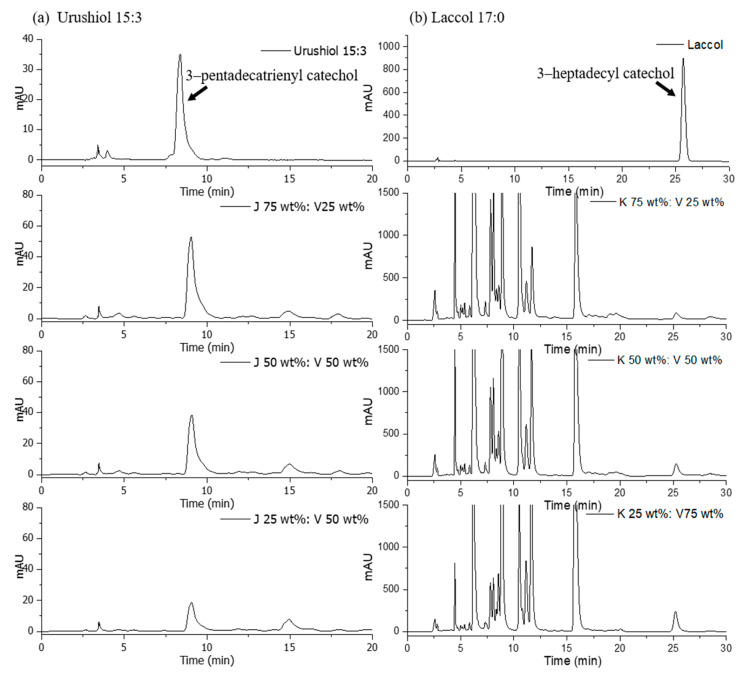
HPLC chromatograms of blended Japanese and Vietnamese lacquers including the following catechols: (**a**) 3-pentadecatrienyl; (**b**) 3-heptadecyl.

**Figure 6:**
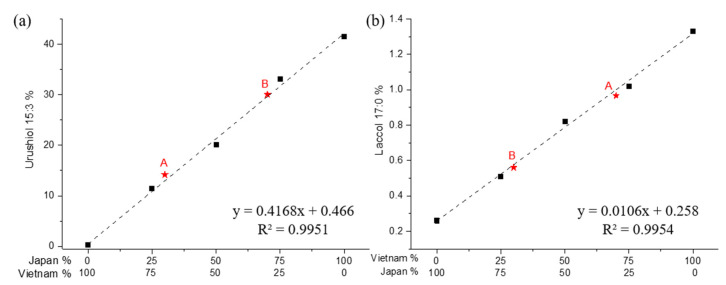
Calibration curves of the compositional ratios of blended Japanese and Vietnamese lacquers as a function of standard catechol peaks using HPLC: (**a**) 3-pentadecatrienyl; (**b**) 3-heptadecyl.

Figure 5 shows HPLC chromatograms of Japanese and Vietnamese lacquer mixtures and 3-pentadecatrienyl catechol and heptadecyl catechol. The ultraviolet spectra of the standards have maxima at the following wavelengths: 3-pentadecatrienyl catechol, 254 nm; and heptadecyl catechol, 210 nm. Chromatograms of urushiol and laccol in the blends were in excellent agreement with standards. Our experiments were sensitive and reproducible. The retention times of 3-pentadecatrienyl catechol and heptadecyl catechol were 9.4 and 25.8 min, respectively. The HPLC chromatograms of the blended lacquers showed characteristic peaks of 3-pentadecatrienyl catechol and heptadecyl catechol. The Japanese and Vietnamese lacquer saps consisted of 42.2 and 0.5 wt% 3-pentadecatrienyl catechol, respectively; and 0.2 and 1.3 wt% heptadecyl catechol, respectively. The calibration curves for pentadecatrienyl catechol (Figure 6a) and heptadecyl catechol (Figure 6b) Japanese/Vietnamese lacquer mixtures were linear. The regression equation for urushiol was y = 0.4168x + 0.466 (R^2^ = 0.9951). For laccol analysis in the blended lacquers, R^2^ was 0.9954. The correlation coefficients indicated good linear relationships between peak height and lacquer ratio over a wide range. Thus, one can use a simple HPLC experiment to quantitatively analyze various lacquers in accordance with the regions in which the lacquer was produced. However, one cannot use HPLC to determine the percent urushiol or laccol quantitatively in the insoluble lacquer films. 

### 3.4. Blind Analysis of Unknown Blended Lacquers

A researcher prepared mixtures of Japanese and Vietnamese lacquers, yet did not identify the exact compositions to the other group of researchers who then performed the analysis to identity the compositions. The blind analysis was performed to verify our quantitative methods. Two different lacquer mixtures were prepared separately and analyzed quantitatively using ToF–SIMS, Py–GC/MS, and HPLC (Figures S3–S5). The asterisks in Figure 2, Figure 4 and Figure 6 show the quantitative results of unknown samples A and B. There was little deviation from the calibration curves (Table 3). ToF–SIMS, Py–GC/MS, and HPLC consistently reported the compositions of unknown lacquer mixtures A and B, prepared as per Japanese:Vietnamese mixture ratios of 3:7 and 7:3, respectively. The use of HPLC based on the content of pentadecatrienyl catechol gave the best quantitative results, having values of 32.3% and 71.1% of Japanese lacquer for the unknown lacquers A and B, respectively.

## 4. Conclusions

We proposed quantitative methods―ToF–SIMS, Py–GC/MS, and HPLC―to determine the ratios of Japanese to Vietnamese lacquers in blends. The blends were polymerized by enzymatic oxidation followed by radical transfer and cross-coupling between urushiol and laccol. We observed several new peaks in the ToF–SIMS spectra of blends. Structural analysis of the dimers indicated that radical transfer occurred between urushiol and laccol derivatives. This is the origin of the dimer structures of coupled catechol derivatives, such as urushiol–urushiol, laccol–laccol, and urushiol–laccol. Characteristic peaks of urushiol and laccol were linear functions of the lacquer ratios, which we used to create a calibration curve for determining percent composition in mixtures. 

We used Py–GC/MS to evaluate the urushiol and laccol contents in blended lacquers. The specific peak intensity as a function of the lacquer composition was linear. HPLC using catechol standards―3-pentadecatrienyl and 3-heptadecyl―provided more-accurate results for Japanese and Vietnamese lacquer blends. HPLC was most efficient and accurate for determining the lacquer ratios, but one can apply ToF–SIMS and Py–GC/MS to quantitative analysis of insoluble lacquer films. Our findings will be useful for evaluating coatings in the Asian lacquer industry and historic remains. 

## Figures and Tables

**Table 1:** Compositional ratios of blended Japanese and Vietnamese lacquers.

Blend	Japanese (wt%)	Vietnamese (wt%)
JV01	100	0
JV02	75	25
JV03	50	50
JV04	25	75
JV05	0	100
Unknown A	30	70
Unknown B	70	30

**Table 2:** Instrumental conditions for ToF-SIMS, Py-GC/MS, and HPLC measurements and standard samples.

**ToF–SIMS**	- Primary ion: Bi_3_^+^ - Energy: 30 keV - Current: 0.6 pA - Analysis area: 100 μm × 100 μm - Polarity: Positive
**Py-GC/MS**	- Injection volume: 10 μL - Furnace: 500 °C; pyrolyzer interface: 300 °C - Oven temperature: 40 °C (2 min) to 320 °C (14 min) at 20 °C/min
**HPLC, urushiol**	- Column: C18 reverse-phase column YMC-Pack Pro C18, 250 × 4.6 mm I.D. S-5 µm, 12 nm - Mobile phase, 90:10 *v*/*v*: Eluent A: Acetonitrile 90% Eluent B: Acqueous trifluoroactic acid, 0.1 vol% - Flow: 1 mL/min - Injection volume: 10 µL - Temperature: 30 °C - Detection: UV, 254 nm - Sample solvent: Chloroform
**HPLC, laccol**	- Column: C18 reverse-phase column YMC-Pack Pro C18, 250 × 4.6 mm I.D. S-5 µm, 12 nm - Mobile phase, 5:95 *v*/*v*: Eluent A: 0.1% THF in dilute water 5% Eluent B: Acetonitrile 95% - Flow: 1 mL/min - Injection volume: 10 µL - Temperature: 40 °C - Detection: UV, 210 nm - Sample solvent: methanol
**3-[(8E,11E)-pentadeca-8,11,14-trienyl] benzene-1,2-diol**	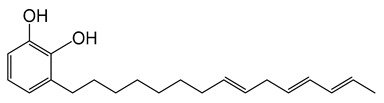
**3-heptadecylbenzene-1,2-diol**	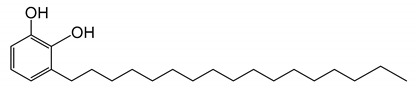

**Table 3:** Quantification of three methods for unknown samples A and B using their calibration curves.

		**ToF–SIMS**	**Py–GC/MS**	**HPLC**	**Used Contents**
		**Jap.:Viet.**	**Jap.:Viet.**	**Jap.:Viet.**	**Jap.:Viet.**
Based on Urushiol	Unknown A	33.4:66.6	32.4:67.6	32.9:67.1	30.0:70.0
	Unknown B	73.4:26.6	71.3:28.7	71.1:28.9	70.0:30.0
Based on Laccol	Unknown A	34.6:65.4	25.2:74.8	33.9:66.1	30.0:70.0
	Unknown B	66.1:33.9	65.9:34.1	71.2:28.8	70.0:30.0

## Data Availability

Data is contained within the article or supplementary material.

## Supplementary Materials

The following are available online at https://www.mdpi.com/2073-4360/13/1/97/s1, Figure S1: Positive-ion ToF–SIMS spectrum of Japanese T. vernififluum lacquer film in the mass range m/z = 0–720.; Figure S2: Positive-ion ToF–SIMS spectrum of Vietnamese T. succedaneum lacquer film in the mass range m/z = 0–720.; Figure S3: Positive-ion ToF–SIMS spectra of unknown lacquer films A and B in the following mass ranges: (a) m/z = 300–370 and (b) m/z = 600–700.; Figure S4: Py-GC/MS total ion chromatograms of unknown lacquers A and B.; Figure S5: HPLC Chromatograms of unknown lacquers A and B based on a) 3-pentadecatrienyl catechol and (b) 3-heptadecyl catechol.
