# Supplementary material for: Quantitative Analysis of Blended Asian Lacquers Using ToF–SIMS, Py–GC/MS and HPLC
Source: Polymers (Basel). 2020 Dec 29;13(1):97. doi: 10.3390/polym13010097 (PMC7795005; doi:10.3390/polym13010097)
Supplement: Supplementary file 1 [file polymers-13-00097-s001.pdf]

Supplementary Information

Quantitative analysis of blended Asian lacquers using ToF-SIMS, Py-GC/MS and HPLC

Hye Hyun Yu[1,2], Jung-Ah Lim[3], Seung Wook Ham[2], Kang-Bong Lee[4], and Yeonhee Lee[1*]

[1]Advanced Analysis Center, Korea Institute of Science and Technology, Seoul 02792, Korea

[2]Department of Chemistry, Chung-Ang University, Seoul 06974, Korea

[3]Post-Silicon Semiconductor Institute, Korea Institute of Science and Technology, Seoul 02792, Korea

[4]National Agenda Research Division, Korea Institute of Science and Technology, Seoul 02792, Korea

*Corresponding author; E-mail: yhlee@kist.re.kr, Tel: +82-2-958-5971, FAX: +82-2-958-5969

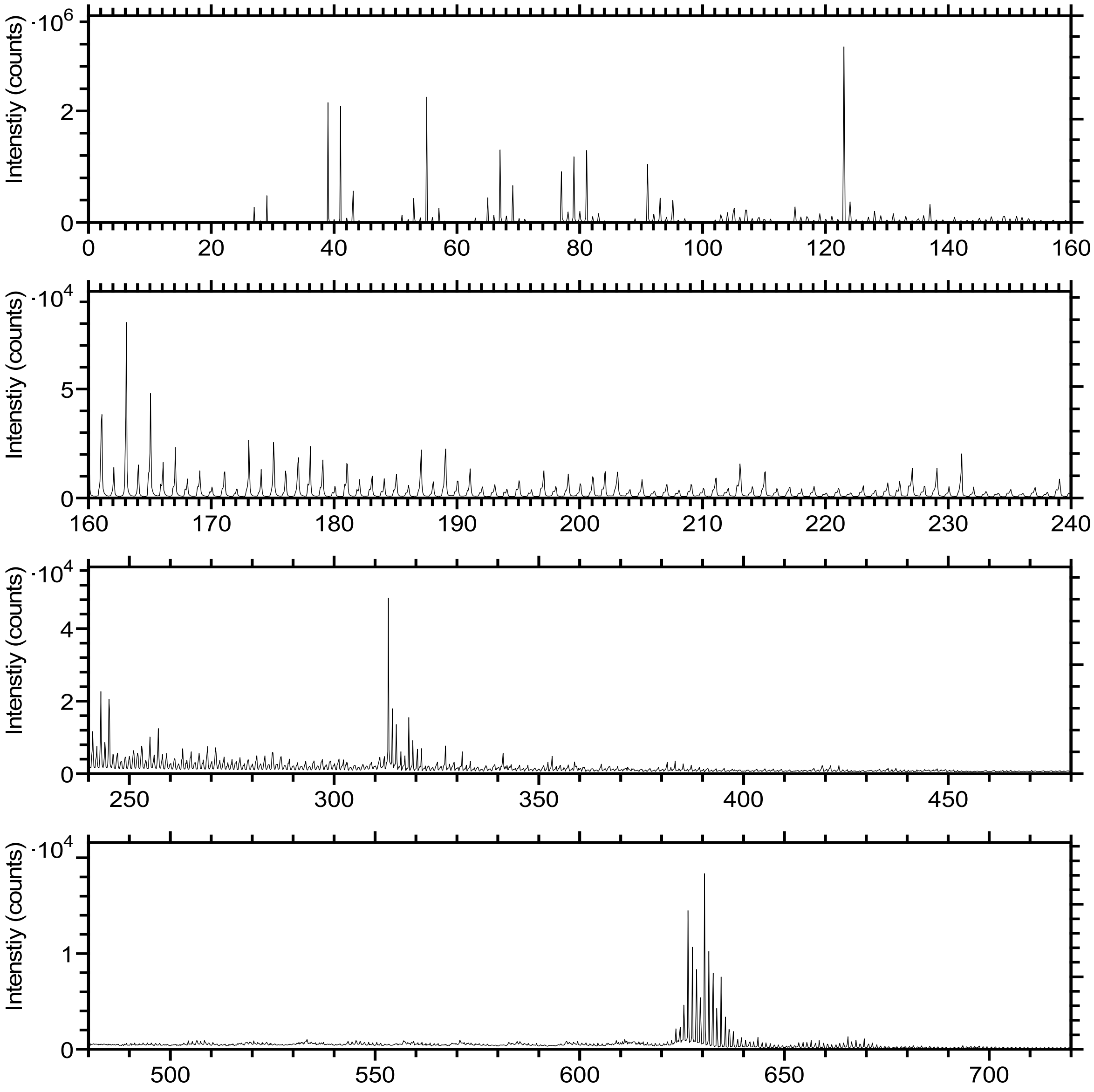

Fig. S1. Positive-ion ToF−SIMS spectrum of Japanese *T. vernififluum* lacquer film in the mass range m/z = 0–720.

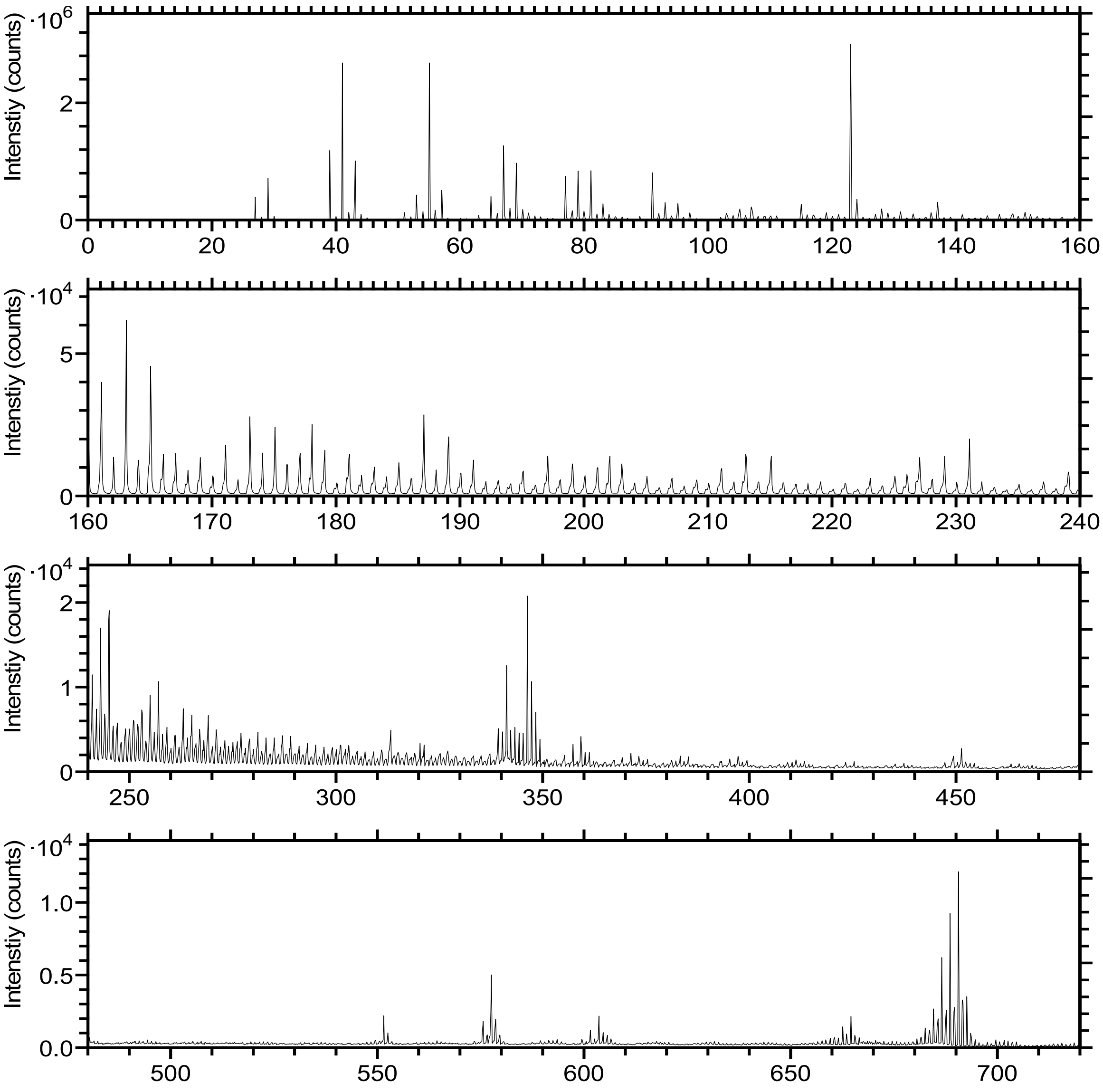

Fig. S2. Positive-ion ToF−SIMS spectrum of Vietnamese *T. succedaneum* lacquer film in the mass range *m/z* = 0−720.

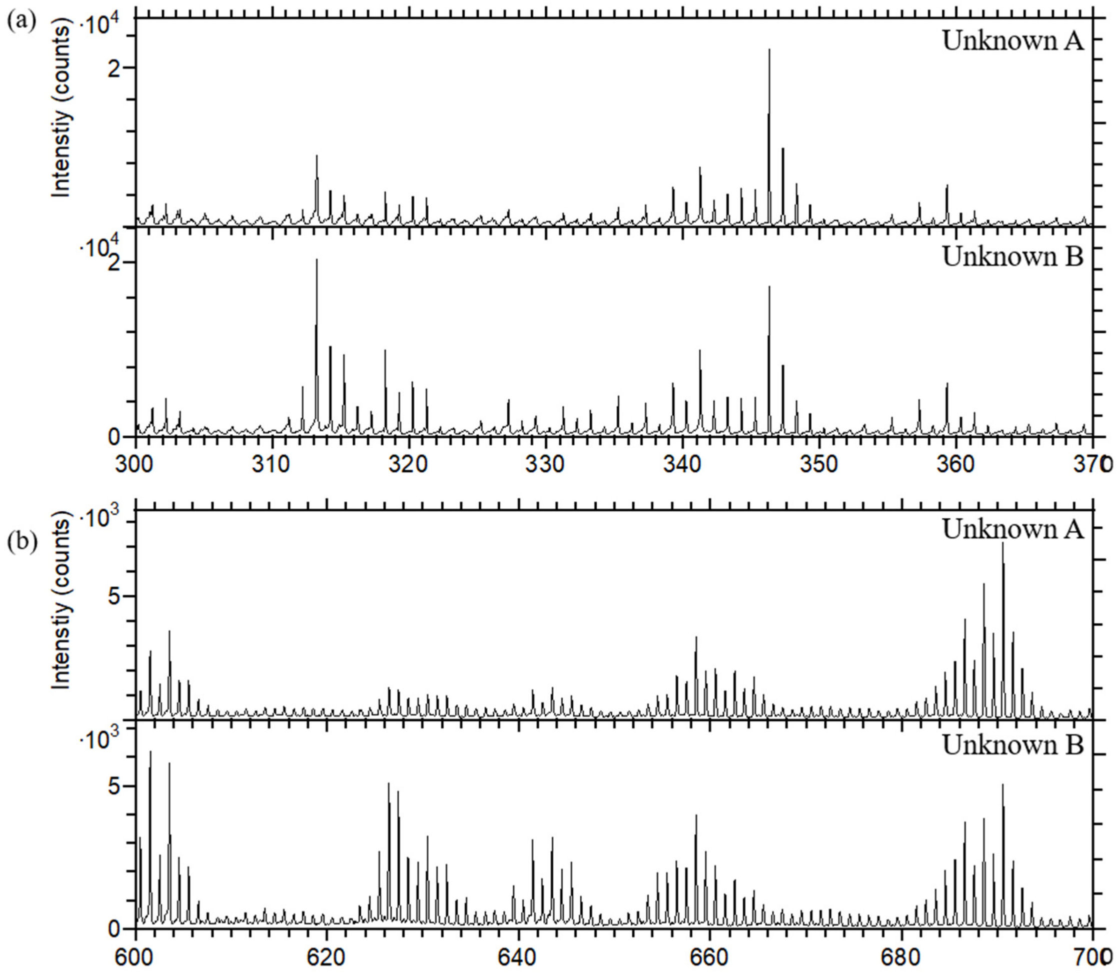

Fig. S3. Positive-ion ToF−SIMS spectra of unknown lacquer films A and B in the following mass ranges: (a) m/z = 300–370 and (b) m/z = 600–700.

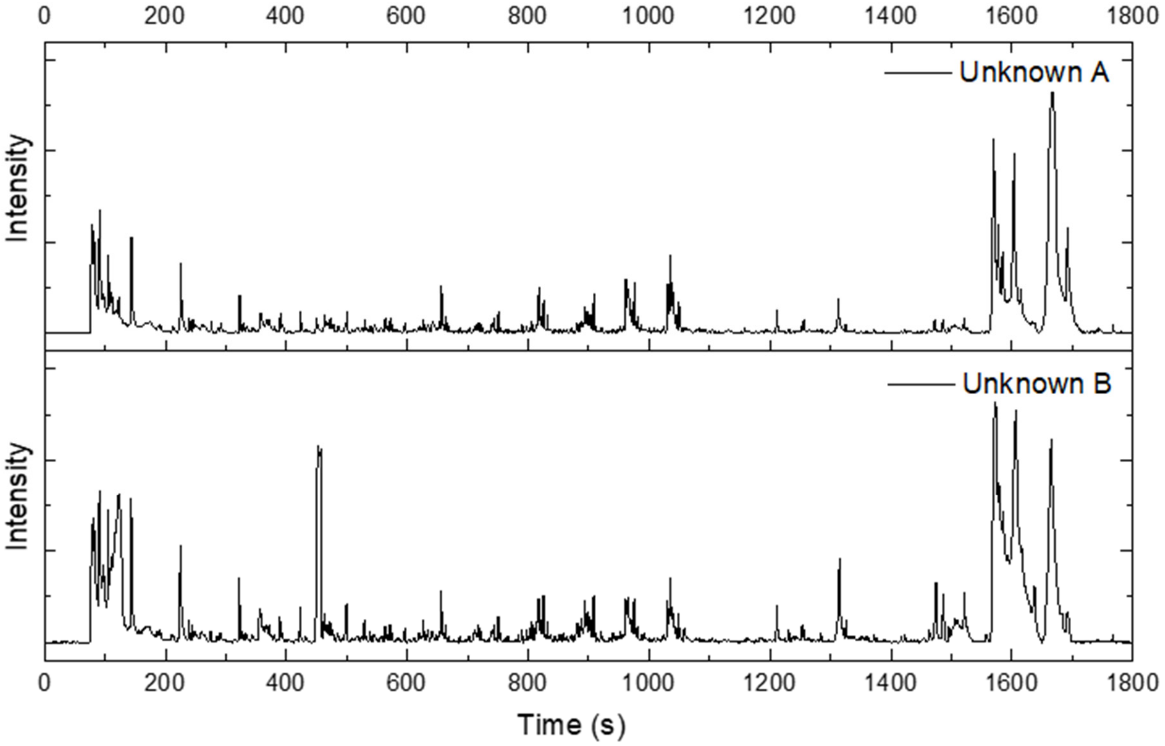

Fig. S4. Py-GC/MS total ion chromatograms of unknown lacquers A and B.

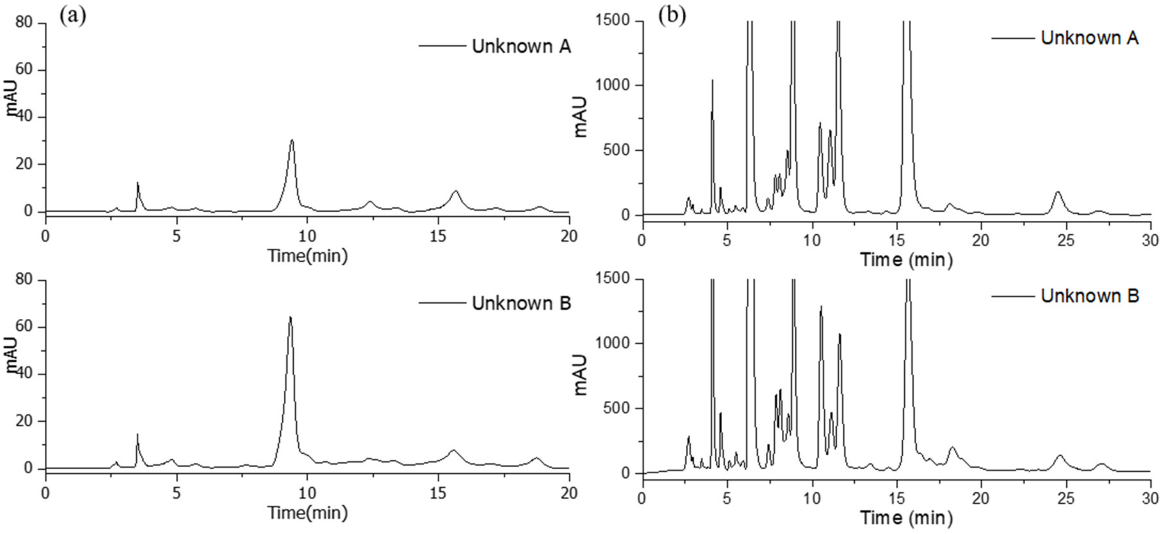

Fig. S5. HPLC Chromatograms of unknown lacquers A and B based on a) 3-pentadecatrienyl catechol and (b) 3-heptadecyl catechol.
